# Association Between Intergenerational Relationship With Adult Children and Anxiety‐Depression Comorbidity Symptoms in Older Women in China: A National Study Using Latent Profile Analysis

**DOI:** 10.1155/da/6040304

**Published:** 2026-06-15

**Authors:** Zishuo Huang, Jiaoqi Ren, Hanlei Tang, Gonghang Qiu, Borui Yang, Ying Wang, Houguang Zhou

**Affiliations:** ^1^ Department of Geriatrics, National Clinical Research Center for Aging and Medicine, Huashan Hospital, Fudan University, Shanghai, 200040, China, fudan.edu.cn; ^2^ The School of Public Health, Fudan University, Shanghai, 200032, China, fudan.edu.cn; ^3^ The Key Laboratory of Public Health Safety, NHC Key Laboratory of Health Technology Assessment, Fudan University, Shanghai, 200032, China, fudan.edu.cn

**Keywords:** anxiety-depression comorbidity symptoms, intergenerational relationship, latent profile analysis, older women

## Abstract

**Background:**

The phenomenon of global aging is becoming increasingly salient, and the mental health of older women requires greater attention. However, there is a conspicuous paucity in the exploration of anxiety‐depression comorbidity patterns among Chinese older women, as well as the correlation between intergenerational relationships with adult children and these comorbid conditions.

**Methods:**

About 20,153 older women aged 65 years or above from the China Aging and Health Survey (CAHS) were included in the study. Depression and anxiety were assessed using the Patient Health Questionnaire‐9 (PHQ‐9) and the 7‐item Generalized Anxiety Disorder (GAD‐7) Questionnaire, respectively. Latent profile analysis (LPA) was utilized to identify subgroups of older women exhibiting similar patterns of symptoms. To examine the association between intergenerational relationships with adult children and the comorbid patterns of anxiety and depression, multinomial logistic regression was employed. Finally, stratified analyses were conducted to perform sensitivity testing.

**Results:**

LPA revealed three profiles: low (*n* = 13,658), moderate (*n* = 5762), and high comorbidity (*n* = 733). Poorer intergenerational relationships with adult children significantly increased odds of moderate (odds ratio [OR] = 1.079, 95% confidence interval [CI]:1.045–1.114) and high comorbidity (OR = 1.187, 95% CI:1.006–1.275) versus the low‐comorbidity group after covariate adjustment. Stratified analyses confirmed robustness.

**Conclusion:**

Anxiety‐depression comorbidity patterns in older women can be classified into three categories: low, moderate, and high comorbidity. Moreover, intergenerational relationships with adult children are associated with these comorbid patterns, with more frequent interactions corresponding to lower levels of comorbidity.

## 1. Introduction

With advancing age, individuals frequently experience escalating disabilities, largely stemming from mental health issues. Depression and anxiety, often comorbid, are prevalent mental health issues that significantly contribute to aging‐related health challenges [1], which can lead to significant psychological, physical, and social impairments, increasing the risk of accelerated brain aging and suicide [1]. Comorbid anxiety and depression are common in older adults, with depressive symptoms reported in up to 90% of anxious elderly patients [2]. Regional surveys in China show that the prevalence of anxiety and depression among older adults ranges from 11.77% to 22.3% and 26.5% to 40.3%, respectively, indicating a high likelihood of co‐occurrence in this population [3].

Older women exhibit a heightened vulnerability to mental disorders relative to their male counterparts, partly due to societal prejudices, reduced occupational awareness, and increased susceptibility to domestic violence [4]. A recent study using data from the Irish Longitudinal Study on Aging (TILDA) cohort aimed to clarify sex differences in the comorbidity of depression and anxiety [2]. The findings revealed that older women were more likely than older men to report comorbidity of depression and anxiety, with a risk difference of 4%. Research in China has also explored the heterogeneity of anxiety and depression comorbidity in widowed older Chinese adults using latent profile analysis (LPA) and network analysis [5]. Concurrently, studies have examined the comorbidity of anxiety and depression among older adults in western China and its ramifications on frailty [6, 7]. However, there remains a paucity of studies exploring the comorbidity patterns of anxiety and depression using LPA among older women in China, with the aim of identifying subpopulations exhibiting similar symptom profiles [8].

Contact with children, integral to the vertical bonds within aging families, underscores a crucial familial support mechanism, enhancing the exchange of instrumental and emotional intergenerational resources [9]. Owing to the traditional gender role divisions and the statistically longer life expectancy [10], which often results in a higher likelihood of women becoming widows, older Chinese women exhibit a significantly greater requirement for intergenerational relationships with their offspring than their male counterparts [11, 12]. Moreover, owing to the relatively low level of social benefits, particularly pensions, in China and the subsequent lack of retirement plans for the majority of older women, many are compelled to rely on the support of their adult children as a safety net [13]. However, in light of explicit filial responsibilities, legal obligations for adult children to support their parents, and the generally modest incomes of most older women, they may harbor the perception of being a burden to their families [14]. Thus, intergenerational relationships with adult children are potentially impactful on the mental well‐being of older Chinese women.

Although existing studies have provided valuable insights into sex differences in anxiety‐depression comorbidity [15, 16], most have included both sexes or focused on gender comparisons, leaving a lack of research specifically targeting older women. Moreover, older Chinese women exhibit unique vulnerabilities that justify a separate, female‐focused investigation. First, due to longer life expectancy, higher widowhood rates, lower pension coverage, and traditional gender roles that emphasize reliance on family, older women depend more heavily on intergenerational relationships with adult children than older men do [17–19]. Second, the impact of intergenerational ties on mental health may operate through distinct pathways in women (e.g., emotional support seeking and perceived burden of caregiving), which could be masked if both sexes are analyzed together [20, 21]. Therefore, excluding men allows us to avoid confounding by gender differences and to provide a clearer, sex‐specific understanding of how intergenerational relationships with adult children are associated with anxiety‐depression comorbidity patterns. However, current studies have not yet explored this relationship between intergenerational ties and co‐occurring anxiety and depression patterns.

Consequently, this research employed LPA to explore variations in anxiety and depression comorbidity among older women and to examine the links between these comorbidity patterns and their intergenerational relationships with adult children. This study is practically valuable as it identifies distinct subgroups with unique symptom profiles, deepens the understanding of anxiety and depression heterogeneity in older women, and enhances knowledge of the complex relationships between intergenerational interactions and mental health outcomes in older Chinese women, thereby contributing to the gerontological and mental health literature.

## 2. Methods

### 2.1. Study Settings

We used data from the China Aging and Health Survey (CAHS) conducted in 2024 by Fudan University HuaShan Hospital. The CAHS is a national survey of Chinese residents aged 65 and older, covering 31 provincial‐level administrative divisions and 434 prefectural‐level cities, districts, and counties (details in the Supporting Information 1). Considering urban/rural stratification and economic levels, along with feasibility and economic validity, a stratified multi‐stage sampling approach was used. This method employed probability proportionate to size sampling (PPS), considering age structure and gender, with random sampling techniques. This ensured that the demographic composition and economic status of the sampled population reflected those of the overall population. Additionally, efforts were made to ensure national representativeness, considering topographical variations and geographical balance.

During on‐site research, a hybrid approach combining offline surveys and online data collection was used to gather seven datasets, including demographic characteristics, health needs, quality of life, geriatric syndrome prevalence, nutritional profiles, motor function, and cognitive‐psychological assessments. The quality control process occurred in three phases: pre‐survey, mid‐survey, and post‐survey (details in Supporting Information 1: Additional File S1). After quality control, 41,859 older adults aged 65 or older were included in the final sample, with all participants or their legal representatives signing informed consent forms. The study was approved by the Fudan University Huashan Hospital Ethics Committee (HIRB2022LS057‐1).

### 2.2. Procedure and Participants

Respondents were excluded if they were men (19,893), did not match the data of marital status (1482), or did not have children (331). The final sample consisted of 20,153 older women from China.

Excluding the 331 older women without offspring was justified due to the high fertility rates among women in 1960s China [13], influenced by factors such as delayed family planning policies, traditional beliefs in the importance of having many children, economic reliance on family labor in rural areas, inadequate social security systems, and limited educational opportunities for women, which reinforced their domestic roles. These factors made having multiple children a norm and a necessity for familial and economic reasons.

### 2.3. Measurements

#### 2.3.1. Intergenerational Relationship

Due to the lack of validated tools, this study adapted a novel questionnaire to explore intergenerational exchanges between older adults and their children based on prior research by Zhong et al. [22]. The CAHS questionnaire was developed by a multidisciplinary team including geriatricians, public health researchers, and survey methodologists, and the frequency of children’s visits (FCV) item was reviewed by this team for face validity and clarity. Participants provided detailed responses, revealing that childlessness among older women was rare, making robust comparisons impossible. Thus, the “childless” option was removed, resulting in seven refined categories: “almost every day” (1), “several times a week” (2), “several times a month” (3), “several times a year” (4), “less than once a year” (5), “years apart, frequent online contact” (6), and “never see or contact” (7). Option 6 (“years apart, frequent online contact”) is distinct from option 7 (“never see or contact”); it is ordered between options 5 and 7 to reflect that, despite the absence of in‐person visits, frequent digital interaction represents a higher level of connection than no contact at all. Higher scores indicated more strained intergenerational relationships.

#### 2.3.2. 7‐Item Generalized Anxiety Disorder (GAD‐7)

Anxiety was assessed using the GAD‐7 scale developed by Spitzer et al. [23]. Participants reported the frequency of each DSM‐IV‐compatible anxiety symptom over the past 2 weeks, choosing from “not at all” (0), “several days” (1), “more than half the days” (2), and “nearly every day” (3). Higher total scores (0–21) indicate more severe anxiety symptoms.

#### 2.3.3. Patient Health Questionnaire‐9 (PHQ‐9)

Depressive symptom severity was assessed using the PHQ‐9, which measures anhedonia, depressed mood, sleep disturbances, fatigue, appetite changes, self‐esteem, concentration, psychomotor agitation/retardation, and suicidal thoughts. Each item was scored on a scale from 0 (“not at all”) to 3 (“almost every day”), resulting in a total score ranging from 0 to 27 [24].

#### 2.3.4. Covariates

The covariates were considered in line with an existing study [5, 25]. Individual‐level covariates included demographic characteristics and health status. Sociodemographic characteristics included age (65–75 years old, 76–85 years old, and >85 years old), marital status (widowed and non‐widowed), education level (literate and illiterate), and residence (urban and rural). Health status included the prevalence of chronic diseases (yes or no) and self‐rated health (SRH).

### 2.4. Statistical Analysis

Rather than using fixed cutoffs (e.g., PHQ‐9 ≥ 3 and GAD‐7 ≥ 3) to define binary comorbidity status, we employed LPA to leverage all item‐level information. LPA identifies empirically distinct subgroups based on symptom patterns, preserves severity gradients, and avoids arbitrary thresholds, thereby providing a more nuanced classification. For the FCV, which is a single ordinal item with a clear hierarchical order, we did not apply LPA, as it is designed for multiple indicators and would not yield additional information beyond the original categories. Instead, we used it directly as an observed predictor.

First, LPA with Mplus 8.3 was used to categorize older women into classes based on their PHQ‐9 and GAD‐7 scores. The 16 items were treated as continuous indicators (0–3 scale), and models were estimated using the robust maximum likelihood estimator (MLR). The analysis started with a single‐profile model and expanded incrementally. Model adequacy was evaluated using the Akaike Information Criterion (AIC), Bayesian Information Criterion (BIC), sample size‐adjusted BIC (aBIC), entropy, bootstrapped likelihood ratio test (BLRT), and the Lo‐Mendell‐Rubin adjusted likelihood ratio test (LMRT). Lower AIC, BIC, and aBIC values indicated a better fit, while significant BLRT and LMRT results confirmed the superiority of the *k*‐class model over the (*k*−1)‐class model. Higher entropy values (approaching one) signified more precise classification. Changes in PHQ‐9 and Generalized Anxiety Disorder (GAD) scores across classes were visualized using a line chart.

Second, descriptive analyses summarized participant characteristics using mean scores, standard deviations, frequencies, and percentages. ANOVA and chi‐square tests compared the features of older Chinese women across the LPA‐identified profiles.

Third, a sankey diagram (origin 2024) visualized comorbidity patterns across the seven categories of FCV, showing the proportions of unique FCV profiles within each comorbidity population.

Fourth, multinomial logistic regression assessed associations between FCV (as a continuous linear term) and anxiety‐depression comorbidity profiles (dependent variable) while controlling for covariates (age, marital status, education, residence, SRH, and chronic diseases). Odds ratios (ORs) and 95% confidence intervals (CIs) were calculated, with *p*  < 0.05 considered statistically significant. Multicollinearity among the independent variables was assessed using the variance inflation factor (VIF), and all VIF values were below 2, indicating no substantial multicollinearity.

Fifth, a sensitivity test used stratification by sociodemographic variables to independently examine associations between FCV and LPA results, adjusting for covariates. These subgroup analyses were conducted as supportive evidence for the main findings, not as formal tests of interaction.

Finally, to test the robustness of the linear assumption for FCV in the primary multinomial logistic regression model, we conducted an additional sensitivity analysis using orthogonal polynomial contrasts. Specifically, FCV was entered into the model as polynomial terms (linear, quadratic, cubic, quartic, quintic, and sextic) to assess whether higher‐order nonlinear trends significantly improved model fit. A joint test (likelihood‐ratio test) was performed for all polynomial terms of order ≥2 (poly2–poly6). A statistically significant joint test would suggest the presence of nonlinear components, whereas a non‐significant joint test would support the linear specification.

## 3. Result

### 3.1. Latent Profile Approach for Older Women in China

This study used LPA with five profiles to investigate anxiety‐depression comorbidity in older women (Table 1). The table lists fit indices, profile quantities, and probabilities. There is no universal standard for fit statistics in LPA methodologies [26]. The optimal model should have lower AIC, BIC, and aBIC values, significant LMR and BLRT (*p*  < 0.05), and entropy > 0.80 [27]. Furthermore, profiles with modest sizes (>2%) were considered viable [28]. The results showed decreasing AIC, BIC, and aBIC values with increasing category counts, favoring the five‐profile model. However, a five‐profile model with 1.64% proportion (<2%) led to its dismissal. The four‐profile model met the minimum class size threshold (2.80%) and showed slightly lower information criteria than the three‐profile model, but its fourth class did not represent a qualitatively distinct symptom profile; instead, it split the moderate or high comorbidity group into two very similar subgroups, offering no additional clinical interpretability beyond the three‐profile solution. Therefore, the four‐profile model was not retained. Significant LMR and BLRT differences confirmed the *K*‐profile model’s superior fit over the (*K*−1)‐profile model. To ensure model stability and avoid local maxima, we used 5000 random starts and 1000 final stage optimizations in Mplus 8.3 for each candidate model; the log‐likelihood values were consistently replicated across multiple runs, indicating no convergence issues. The three‐profile model, with perfect entropy (1), was deemed more suitable and provided insightful psychological outcome categorization [29]. Consequently, the three‐profile model was chosen as the definitive model, delineating the profiles into low, moderate, and high comorbidity categories.

**Table 1:** Optimal model fit evaluations for latent profile analysis models with 1–5 classes (*n* = 20,153).

LPA models	AIC	BIC	aBIC	LMRT(*p*‐value)		BLRT(*p*‐value)	Entropy	Smallest profile (%)
				Value	*p*‐Value			
1‐Profile	598243.035	598496.184	598394.489	—	—	—	—	—
2‐Profile	409426.341	409813.975	409658.255	187736.435	<0.001	<0.001	0.988	30.10
3‐Profile	326232.999	326755.119	326545.374	82736.282	0.0027	<0.001	1.000	3.64
4‐Profile	305546.837	306203.443	305939.673	20610.709	<0.001	<0.001	0.992	2.80
5‐Profile	284282.552	278514.586	278196.79	27717.509	<0.001	<0.001	1.000	1.64

**Figure 1:**
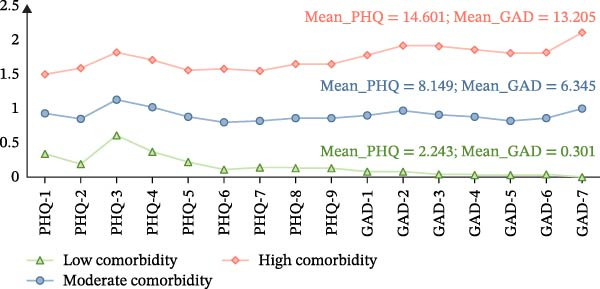
Analytical profile plot encapsulating the item scores of PHQ‐9 and GAD‐7 across the three‐class model spectrum.

Figure 1 shows the means of 16 items related to anxiety and depressive symptoms across three profiles. Significant differences exist among the means, indicating distinct characteristics. The “low comorbidity” profile had the lowest means (PHQ‐9:2.243 and GAD‐7:0.301), the “moderate comorbidity” profile had higher scores (PHQ‐9:8.149 and GAD‐7:6.345), and the “high comorbidity” profile had the highest scores (PHQ‐9:14.601 and GAD‐7:13.205).

### 3.2. Characteristics of Study Participants Based on LPA

The dataset included 20,153 older Chinese women, mostly aged 65–75 (65.07%), non‐widowed (75.42%), literate (70.95%), urban residents (62.62%), and with chronic diseases (76.59%). The average SRH and FCV scores were 2.77 (SD = 0.991) and 1.80 (SD = 0.993). Chi‐square analysis showed that older women who were older, widowed, illiterate, rural residents, and with chronic diseases were more likely to have higher anxiety‐depression comorbidity. Conversely, ANOVA indicated that better SRH and intergenerational relationships were associated with lower anxiety‐depression comorbidity (Table 2).

**Table 2:** Baseline characteristics in terms of the profiles of anxiety‐depression comorbidity symptoms among older women in China (*n* = 20,153).

Variables	Low comorbidity (*n* = 13658/67.78%) Mean ± SD/N (%)	Moderate comorbidity (*n* = 5762/28.60%) Mean ± SD/N (%)	High comorbidity (*n* = 733/3.64%) Mean ± SD/N (%)	Total	*X* ^2^	*p*‐Value
Age					100.00	<0.001
65–75 years old	9166 (67.11%)	3531 (61.28%)	416 (56.75%)	13,113 (65.07%)	—	—
76–85 years old	3502 (25.64%)	1714 (29.75%)	219 (29.88%)	5435 (26.97%)	—	—
>85 years old	990 (7.25%)	517 (8.97%)	98 (13.37%)	1605 (7.96%)	—	—
Marital status					5.98	<0.05
Widowed	3292 (24.11%)	1465 (25.43%)	197 (26.88%)	4954 (24.58%)	—	—
Non‐widowed	10,366 (75.89%)	4297 (74.57%)	536 (73.12%)	15,199 (75.42%)	—	—
Education level					169.35	<0.001
Literate	10,063 (73.67%)	3805 (66.04%)	431 (58.80%)	14,299 (70.95%)	—	—
Illiterate	3595 (26.33%)	1957 (33.39%)	302 (41.20%)	5854 (29.05%)	—	—
Residence					131.12	<0.001
Urban	8894 (65.11%)	3358 (58.28%)	368 (50.20%)	12,620 (62.62%)	—	—
Rural	4764 (34.89%)	2404 (41.72%)	365 (49.80%)	7533 (37.38%)	—	—
Chronic disease					335.03	<0.001
Yes	9958 (72.91%)	4817 (83.60%)	660 (90.04%)	15,435 (76.59%)	—	—
No	3700 (27.08%)	945 (16.40%)	73 (9.96%)	4718 (23.41%)	—	—
SRH	2.925 ± 0.997	2.483 ± 0.891	2.115 ± 0.825	2.77 ± 0.991	1358.94	<0.001
FCV	1.770 ± 0.978	1.854 ± 1.010	1.963 ± 1.095	1.80 ± 0.993	75.39	<0.001

Abbreviations: FCV, frequency of children’s visits; SRH, self‐reported health.

### 3.3. The Result of Sankey Diagram

**Figure 2:**
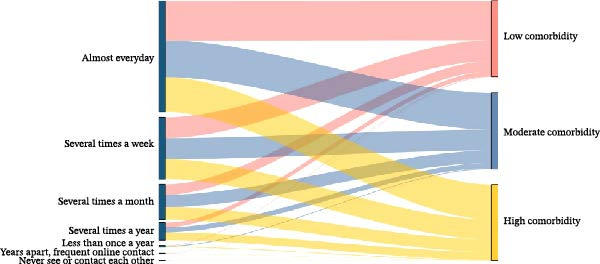
A sankey diagram showcasing the overlap between latent profile analysis results on anxiety‐depression comorbidity symptoms and the frequency of children’s visits among older women.

Figure 2 presents a sankey diagram illustrating the association between FCV and anxiety‐depression comorbidity profiles in older women. The left side shows the seven FCV categories, and the right side shows the three comorbidity profiles (low, moderate, and high). The colored bands represent the distribution of comorbidity patterns across FCV categories. As comorbidity severity increased, the proportion of daily child visitation decreased (low: 51.96%, moderate: 48.14%, and high: 45.57%), whereas the proportion of yearly visits increased (low: 6.17%, moderate: 6.66%, and high: 10.37%) (detailed data was presented in Supporting Information 2: Additional File S2).

### 3.4. Results of Multinomial Logistic Regression

**Figure 3:**
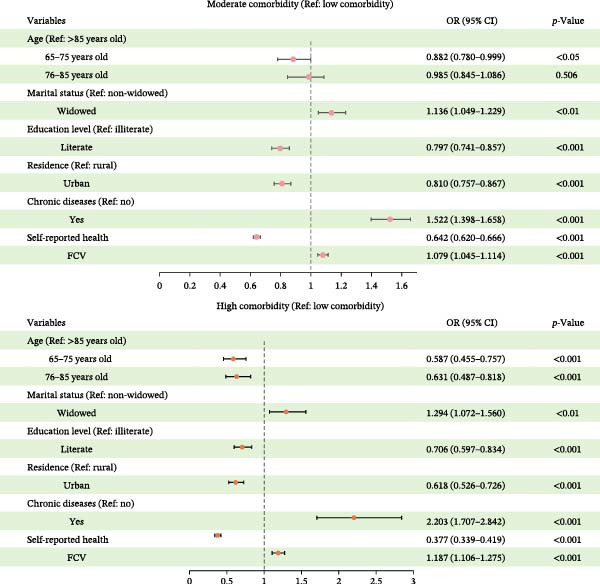
Multinomial logistic regression analysis of different latent profile.

After adjusting for covariates, older women with poorer intergenerational relationships (OR: 1.079, 95% CI: 1.045–1.114; OR: 1.187, 95% CI: 1.006–1.275) were more likely to be classified as having moderate or high comorbidity compared to those with low comorbidity. Other factors reducing the likelihood of moderate or high comorbidity included being aged 65–75 years (OR: 0.882, 95% CI: 0.780–0.999; OR: 0.587, 95% CI: 0.455–0.757), not widowed (OR: 1.136, 95% CI: 1.049–1.229; OR: 1.249, 95% CI: 1.072–1.560), literate (OR: 0.797, 95% CI: 0.741–0.857; OR: 0.706, 95% CI: 0.597–0.834), residing in urban areas (OR: 0.810, 95% CI: 0.757–0.867; OR: 0.618, 95% CI: 0.526–0.726), having no chronic diseases (OR: 1.522, 95% CI: 1.398–1.658; OR: 2.203, 9 details 2E high comorbidity, respectively. More details are presented in Figure 3. VIF values for all covariates were below 2, confirming the absence of significant multicollinearity (Supporting Information 3: Additional File S3).

### 3.5. Results of Sensitivity Analysis

Sensitivity analyses were performed using stratified methods. The correlation between intergenerational relationships and the comorbidity of anxiety and depressive symptoms in older women was examined via stratification based on age groups (65–75, 76–85, and >85 years), education level (illiterate vs. literate), marital status (widowed vs. non‐widowed), and domicile location (urban vs. rural). Our findings revealed that the regression outcomes across various strata were generally consistent with the main results, providing supportive evidence for the primary association rather than suggesting effect modification more details are presented in the Table 3.

**Table 3:** Results of sensitivity analysis.

Independent variable	Age			Marital status		Education level		Residence	
	65–75 years old	76–85 years old	>85 years old	Widowed	Non‐widowed	Literate	Illiterate	Urban	Rural
Ref: low comorbidity									
Moderate comorbidity	1.503 (1.012–1.094) ^∗^	1.124 (1.057–1.195) ^∗∗∗^	1.188 (1.045– 1.350) ^∗∗^	1.130 (1.055– 1.209) ^∗∗∗^	1.068 (1.030–1.107) ^∗∗∗^	1.072 (1.031–1.114) ^∗∗∗^	1.098 (1.039 –1.161) ^∗∗^	1.067 (1.022–1.113) ^∗∗^	1.103 (1.051–1.157) ^∗∗∗^
High comorbidity	1.135 (1.035–1.244) ^∗∗^	1.202 (1.053– 1.372) ^∗∗^	1.538 (1.244– 1.902) ^∗∗∗^	1.381 (1.203–1.585) ^∗∗∗^	1.133 (1.043–1.230) ^∗∗^	1.197 (1.091–1.313) ^∗∗∗^	1.184 (1.080–1.299) ^∗∗^	1.281 (1.160–1.414) ^∗∗∗^	1.116 (1.008–1.235) ^∗^

*Note*: The results presented the association between frequency of children’s visit and anxiety‐depression comorbidity symptoms in Chinese older women after adjusting the covariates (age, marital status, education level, residence, chronic diseases, and self‐reported health) in different subgroups.

^∗^ *p* < 0.05.

^∗∗^ *p* < 0.01.

^∗∗∗^ *p* < 0.001.

Further sensitivity analysis using orthogonal polynomial terms showed a strong and significant linear effect of FCV for both comorbidity groups (moderate and high). Individually, nonlinear terms were only sporadically significant: the quadratic (RRR = 0.96) and sextic (RRR = 1.06) terms reached significance solely in the moderate comorbidity group, with no consistent pattern across terms. No nonlinear effects were observed in the high comorbidity group. The linear trend was dominant throughout (Supporting Information 4: Additional File S4).

## 4. Discussion

This study, the first to examine the association between intergenerational relationships with adult children and comorbidity patterns of anxiety and depression in older women using LPA with nationally representative data. An optimal three‐profile model was determined to best describe the data, categorizing 67.78% of the participants into a low comorbidity profile, 28.60% into a moderate comorbidity profile, and 3.64% into a high comorbidity profile. After controlling for covariates, multivariate logistic regression showed that intergenerational relationships with children, particularly increased visitation frequency, were strongly associated with lower comorbidity levels of anxiety and depression in older women. Sensitivity analyses confirmed these findings across diverse socio‐demographic groups, affirming the robustness of the results.

Existing research on anxiety‐depression comorbidity in older adults has largely focused on gender differences in prevalence or symptom scores [2, 30, 31]. For instance, a Canadian study reported that women are twice as likely as men to report lifetime PTSD, which may persist into old age and shape distinct comorbidity patterns [31]. Similarly, British scholars have attributed gender differences to variations in marital quality and socialization patterns [2]. However, most of these studies have not specifically targeted older women, and few have employed LPA to dissect the heterogeneity of anxiety‐depression comorbidity within this population.

This study used LPA to identify subgroups of older women with similar symptom profiles, aiding targeted treatment. In China, heterogeneity in anxiety‐depression comorbidity among older women is driven by low earnings, overly optimistic life expectancy assumptions, and high familial responsibilities. First, Chinese older women often receive inadequate social welfare, with urban women facing a high poverty risk (41.1% extreme poverty) and 65.5% of rural women living on less than 93.9 yuan per month [13]. Second, A significant majority of older women experience widowhood due to their longer life expectancy and younger age relative to their husbands, often leading to living alone, reduced social support, and increased feelings of depression and anxiety [10]. Third, older women tend to bear the responsibility of caring for their families, particularly their grandchildren, which not only prevents them from receiving care from their families but also imposes additional burdens on them, resulting in anxiety and depression [32].

Our findings extend previous literature on intergenerational relationships and mental health in older adults. Prior studies have consistently shown that more frequent contact with adult children is associated with fewer depressive or anxiety symptoms [18, 21, 33, 34]. However, most of these studies used mixed‐sex samples, examined depression and anxiety separately, and did not focus on comorbidity patterns. For example, Chinese studies have reported a negative dose‐response relationship between contact frequency and depressive symptoms [35], and a recent LPA study identified anxiety‐depression comorbidity patterns in general or widowed older adults [5]. Nevertheless, evidence specifically targeting older women remains scarce. To our knowledge, this is the first study to focus exclusively on older Chinese women, use LPA to identify three comorbidity profiles (low, moderate, and high), and examine graded associations with intergenerational relationship frequency in a large national sample (*n* = 20,153). We observed that the magnitude of the association between frequent contact and lower comorbidity was larger for high comorbidity than for moderate comorbidity—a finding not reported in prior mixed‐sex or single‐disorder studies. This study provides sex‐specific, pattern‐based descriptive evidence that intergenerational ties are associated with different levels of anxiety‐depression comorbidity in older women.

Intergenerational relationships between older adults and their children differ between Western and Asian countries. In many Western contexts, supporting parents is more often viewed as a choice rather than an obligation, with parents often relying on pensions or social security [36]. In Western countries like the US, Canada, and many European nations, “nuclear family” structures prevail, with adult children living independently after marriage or adulthood, and elderly parents not cohabiting with their children [37]. In contrast, in Asian countries like China, Japan, and Singapore, filial piety is a core value, and children’s responsibility to support their parents is deeply ingrained [38–40]. Children are expected to care for elderly parents, especially if they cannot care for themselves. Traditional family structures in these countries are multigenerational, known as “extended families,” with parents, adult children, and grandchildren living together [41]. In China, there are marked disparities in intergenerational relationships with children based on gender [11]. Research indicates that Chinese older women, compared to their male counterparts, typically reciprocate with more support from their offspring [12]. One interpretation suggests that sustained gender roles and traditional Confucian principles, promoting ideals like “three obediences and four virtues” for women, foster a heightened sense of reliance and empathy among children towards their mothers [11]. Alternatively, it is plausible that contrasted with older males, older females exhibit a greater propensity to articulate and share their emotions with others, evincing a higher comfort level in seeking out both formal and informal support mechanisms, and adeptly fostering informal social networks [42].

The results of this study revealed a substantial correlation between intergenerational ties with offspring and the comorbidity of anxiety and depression among older women, demonstrating that an increased frequency of visits from children is associated with a reduced severity of comorbid conditions. However, due to the cross‐sectional design, causality cannot be inferred; the observed associations may be bidirectional or confounded by unmeasured factors. This result can be elucidated by the “buffering mechanism” theory [43]. The buffering mechanism theory postulates that both instrumental and emotional social support can act as a buffer zone, mitigating the impact of stressors (e.g., loneliness and health concerns) on adverse health outcomes (e.g., anxiety and depression) [44]. Complementing this, intergenerational solidarity theory [45] emphasizes that contact frequency (associational solidarity) is only one dimension of parent‐child ties [46]; future research should incorporate affectional and functional dimensions. Role theory further suggests that older women, whose social identity is often tied to family roles, may derive a greater psychological benefit from intergenerational contact than men. Our finding of a larger difference in magnitude for high comorbidity than that for moderate comorbidity aligns with this theoretical perspective.

Above all, instrumental support can significantly alleviate comorbid anxiety and depression among Chinese older women [47, 48]. In traditional family structures, older women often serve as the family nucleus and caregivers, with heightened dependency and expectations on their families. Adult children can assist with household chores, shopping, daily routines, accompany medical appointments, remind medication intake, and arrange health check‐ups, thereby facilitating better health management for older women. In discussing emotional support, older women typically exhibit greater emotional requirements compared to their male counterparts [49]. They demonstrate proficiency in building and sustaining social networks, seeking emotional gratification through interpersonal engagements. Online forms of contact—such as video calls, instant messaging, and social media—have become increasingly common ways to maintain intergenerational ties, especially when geographic distance limits in‐person visits. The emotional support and companionship from their children, whether delivered face‐to‐face or online, fulfill these needs, enhancing their psychological well‐being. Regular visits and company from children help alleviate internal stress and anxiety, reducing feelings of loneliness and providing emotional reassurance. Additionally, children’s engagement helps older women preserve and expand their social circles through family reunions, neighborhood activities, and other social events, strengthening social ties and reducing social isolation.

For older women with varying anxiety and depression levels, the following considerations may be drawn from this study: (1) micro level‐personalized mental health support (e.g., education and cognitive‐behavioral therapy) and intergenerational family activities (e.g., interactive activities and digital tools) could be considered [50]. (2) Meso level‐community mental health centers, intergenerational digital platforms, and support groups, along with the use of volunteers and resources, represent potential approaches [51]. (3) Macro level‐policies reinforcing filial obligations, improved mental health insurance, funded community services, and societal efforts to promote respect for older women and intergenerational harmony might be explored [52]. Multilevel coordination may contribute to better mental health outcomes and more harmonious intergenerational relationships.

## 5. Conclusion

In summary, this study identified three distinct anxiety‐depression comorbidity profiles among older Chinese women using LPA: low (67.78%), moderate (28.60%), and high (3.64%). More frequent intergenerational contact with adult children was associated with lower comorbidity levels, and this association was stronger for the high comorbidity profile than for the moderate one. These findings indicate a close link between intergenerational relationships and the severity of anxiety‐depression comorbidity in Chinese older women.

## 6. Limitation

The study has several limitations that need attention and refinement in future research. First, the cross‐sectional design at a single time point limits the examination of symptom changes over time. Future research should use latent transition analysis with longitudinal data to better understand anxiety and depression trajectories. Second, our nationally representative data did not adjust for regional differences (e.g., Eastern, Central, and Western China). Given regional variation in family structures, support norms, and mental health services, the observed associations may be confounded or modified. Future studies should include regional covariates or stratified analyses to address geographic heterogeneity. Third, although we performed stratified analyses by age, education, marital status, and residence as supportive sensitivity checks, we did not conduct formal interaction tests. Interaction tests would provide a more rigorous evaluation of whether the association between FCV and comorbidity differs significantly across subgroups. Therefore, our stratified results should be interpreted cautiously, and future studies should include such tests to assess effect modification. Fourth, using only the FCV as a proxy for intergenerational relationships is limited, as frequency does not equal quality and a linear association cannot be assumed; future research should include multidimensional measures of relationship quality (e.g., emotional support and conflict). Fifth, our study only included older women who had adult children, so the results may not be generalizable to childless or unmarried older women. Future studies should explore how alternative sources of intergenerational support (e.g., siblings, friends, and community networks) are associated with mental health in these populations. Sixth, compared with traditional cutoff‐defined binary comorbidity, LPA provides a more nuanced classification that distinguishes moderate from high comorbidity, revealing a graded association with intergenerational contact. Future studies may compare the predictive validity and clinical utility of LPA‐derived versus cutoff‐based classifications.

## Author Contributions

Conceptualization: Zishuo Huang, Houguang Zhou, and Ying Wang. Formal analysis: Zishuo Huang and Jiaoqi Ren. Methodology: Zishuo Huang and Hanlei Tang. Supervision: Zishuo Huang, Jiaoqi Ren, and Hanlei Tang. Visualization: Zishuo Huang, Jiaoqi Ren, Hanlei Tang, Borui Yang, Gonghang Qiu, Houguang Zhou, and Ying Wang. Writing – original draft: Zishuo Huang. Writing – review and editing: Zishuo Huang, Jiaoqi Ren, and Ying Wang.

## Funding

This study was supported by the National Key R&D Plan “Intergovernmental International Science and Technology Key Special Project”(Grant 2021YFE0111800) and the Noncommunicable Chronic Diseases‐National Science and Technology Major Project (Grant 2024ZD0524200).

## Disclosure

All authors read and approved the final manuscript.

## Ethics Statement

The CAHS was approved by the Fudan University Huashan Hospital Ethics Committee (HIRB2022LS057‐1). All the respondents offered a written consent before participating the survey. Moreover, illiterate and uneducated older adults with disabilities completed written consent in the company of a legally representative third party (children, brothers, sisters, or other member of family).

## Consent

The authors have nothing to report.

## Conflicts of Interest

The authors declare no conflicts of interest.

## Supporting Information

Additional supporting information can be found online in the Supporting Information section.

## Supporting information


**Supporting Information 1** Additional File S1: File of Population coverage and data resource area.

**Supporting Information 2** Additional File S2: The original data of Sankey diagram.

**Supporting Information 3** Additional File S3: Variance inflation factor (VIF) for covariates in the multinomial logistic regression model.

**Supporting Information 4** Additional File S4: Sensitivity analysis: Orthogonal polynomial multinomial logistic regression of comorbidity classes (moderate/high vs. low) on FCV—testing linearity assumption.

## Data Availability

The data that support the findings of this study are available from the corresponding author upon reasonable request.
