# Supplementary material for: Association Between Intergenerational Relationship With Adult Children and Anxiety‐Depression Comorbidity Symptoms in Older Women in China: A National Study Using Latent Profile Analysis
Source: Depress Anxiety. 2026 Jun 15;2026:6040304. doi: 10.1155/da/6040304 (PMC13269846; doi:10.1155/da/6040304)
Supplement: Supplementary file 1 — Supporting Information 1 Additional File S1: File of Population coverage and data resource area. [file DA-2026-6040304-s002.docx]

**1. Population coverage and Data resource area**

**1.1 Sample Subjects**

The target population for this monitoring initiative encompasses community-dwelling Chinese citizens aged 65 and above residing across various administrative levels (provinces/municipalities, cities, counties, districts) in China, excluding those collectively residing in military bases, hospitals, prisons, nursing homes, dormitories, and similar establishments. The focus is on the resident population, defined as individuals who have cumulatively resided in the local area for more than six months within the past 12 months, irrespective of their local household registration status. This includes elderly individuals in both urban and rural communities. Special attention is directed towards specific subgroups such as elderly individuals living alone, those with disabilities, and empty-nest elderly, ensuring their comprehensive inclusion in the scope of the survey.

**1.2 Sample Area**

The sample comprises 31 provincial-level administrative divisions throughout China, encompassing 178 districts, and counties. From each district/county, 2 communities are selected for surveying, excluding the regions of Hong Kong, Macao, and Taiwan. More details can be presented in Figure 1. This nationwide monitoring initiative is specifically tailored to the elderly population, which is categorized into six major regions:

1. North-East: consisting of Liaoning, Jilin, and Heilongjiang.
2. North: including Beijing, Tianjin, Hebei, Shanxi, Inner Mongolia.
3. East: Shanghai, Jiangsu, Zhejiang, Anhui, Fujian, Jiangxi, Shandong.
4. South-Central: Henan, Hubei, Hunan, Guangdong, Guangxi, Hainan.
5. South-West: Sichuan, Guizhou, Yunnan, Tibet, Chongqing.
6. North-West: Shanxi, Gansu, Qinghai, Ningxia, Xinjiang.


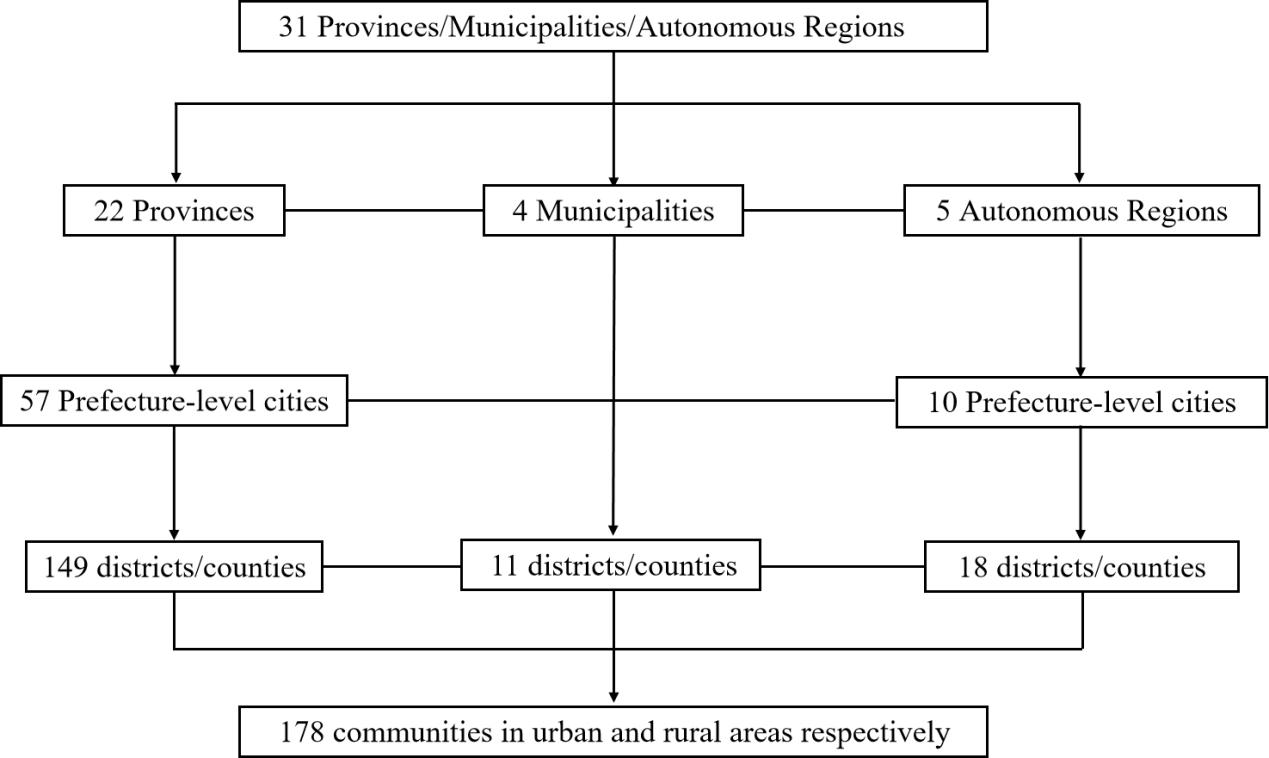


Figure 1. Sample areas

This extensive geographical coverage ensures a representative and comprehensive assessment of the elderly population across diverse regional demographics and socio-economic landscapes.

**1.3 Sample Methods**

Taking into account urban/rural stratification and economic levels, while considering feasibility and economic validity, a stratified multi-stage sampling approach was employed. This method integrated a whole Probability Proportionate to Size Sampling (PPS method) to the population size, considering factors like age structure and gender, with a random sampling technique. This combination ensured that the demographic composition and economic status of the sampled population aligned closely with that of the overall population. Additionally, care was taken to ensure that the samples drawn from the monitoring sites were nationally representative, taking into account topographical variations and geographical balance.

**1.4 Investigation tool**

Fudan University Huashan Hospital, the Shanghai International Institute for Human Phenome Research,, Public Health School of Fudan University, and Feishen Medical Technology Company have collaborated to independently innovate and design a scientifically robust and rational intelligent platform tailored to investigate the health needs of the elderly—the China Elderly Health Network. This platform integrates methodologies of both online operations and offline surveys to disseminate surveys, thereby collecting comprehensive data encompassing the fundamental demographics, health status, lifestyle habits, and social engagement of the elderly population.

**1.5 Sampling Steps**

**Step 1:** The subject expert group selects the lead unit of each province/municipality directly under the central government nationwide and signs a cooperation agreement; Subsequently, 1-2 coordinators were appointed for each province or municipality directly under the central government to undertake the tasks of resource coordination and alignment.

**Step 2:** The quantity of surveyed individuals in each province and municipality directly under the Central Government is ascertained according to the standardized age - group distribution and sex ratio of the elderly population in China.

**Step 3:** Select 2-3 representative cities (1 for high, 1 for low, 1 for high and 1 for low GDP rankings) to be surveyed according to the GDP rankings of cities above the prefecture level in each province: for municipalities directly under the central government, select 2 urban areas and 2 suburbs, and the number of people to be surveyed in each city/district/county will be divided equally according to the total number of people surveyed in the whole province (i.e., 1/3 for each of the high and low GDP rankings, or 1/2 for each of the high and low GDP rankings);

**Step 4:** Each prefectural-level city selects at least 2 districts/counties with medium level of development (1 urban area + 1 suburb/county, with the secretary of the general subject being responsible for providing the candidate list), and each district selects 2 communities to be surveyed, so that a total of 8-12 communities or above in each province are selected to participate in the survey.

**1.6 Quality Control**

The quality control framework for the investigation into the health needs of the elderly is segmented into three phases: pre-survey, mid-survey, and post-survey (Figure 1). Pre-survey, expert consultations are undertaken to establish the research objectives and validate the appropriateness and professional rigor of the survey instruments. During-survey, efforts are made to ensure that the sample population accurately completes the questionnaires in accordance with their personal circumstances, with specialized staff on hand to provide guidance. Post-survey, a data quality assurance process involves telephone follow-ups, where individuals exhibiting anomalies or logical inconsistencies in their responses are identified and contacted separately for clarification.


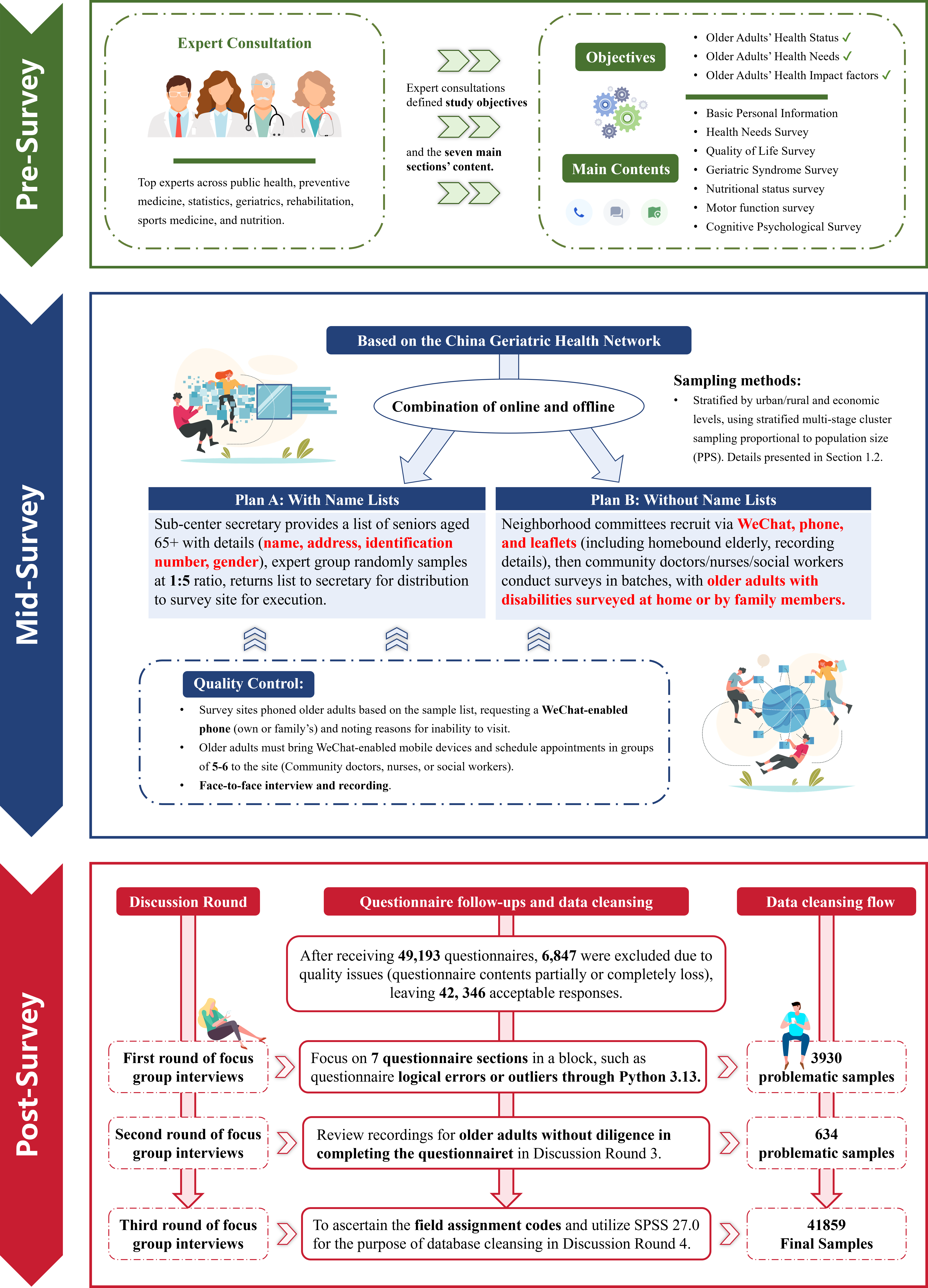


Figure 1. Quality control framework for CAHS

**1.6.1 Pre-survey**

Following numerous in-depth and meticulous project center group discussions, our team assembled top-tier experts from a wide array of disciplines including joint public health, preventive medicine, statistics, geriatrics, rehabilitation medicine, sports medicine, and nutrition. Through multiple rounds of brainstorming sessions, we finalized the objectives of the comprehensive health survey for older adults and delineated the seven primary sections of the questionnaire (Basic Personal Information, Health Needs Survey, Quality of Life Survey, Geriatric Syndrome Survey, Nutritional status survey, Motor function survey, Cognitive Psychological Survey).

**1.6.2 Mid-survey**

To enhance the efficiency and quality of survey data collection, we adopted a hybrid online completion and offline survey methodology based on PPS sampling methods. Online, we leveraged the intelligent platform of the “China Elderly Health Network” WeChat mini-program, which enables participants to conveniently self-complete questionnaires and upload health data. Offline, professionally trained surveyors conducted face-to-face interviews to assist in filling out electronic questionnaires, particularly for participants with lower educational attainment or difficulties using electronic devices. This comprehensive approach ensures the completeness and accuracy of data collection.

During the survey process, we implemented two methodologies, designated as Plan A and Plan B:

**Plan A:**

The sub-center project secretaries provide a list of individuals aged 65 and older within the sampled communities, including their names, genders, ages, addresses, and contact information (recommended to be within 3 kilometers of the survey site). The expert panel then conducts a random sampling at a ratio of 1:5 and provides the results back to the sub-center project secretaries, who subsequently distribute the list to the survey sites for implementation.

**Plan B:**

Recruitment is managed by the neighborhood committee, street office, or community health center. They recruit participants through various channels such as WeChat groups (including groups for signed residents, neighborhood committees, and building leaders), telephone calls, and informational flyers. This recruitment includes homebound, disabled, and bedridden elderly individuals, for whom demographic details (names, genders, ages, addresses, and contact information) are recorded. Community doctors, nurses, or social workers then conduct the surveys in batches. For disabled elderly individuals, the surveys can be completed either through home visits or by their family members on their behalf.

**1.6.3 Post-survey**

After administering 49,193 questionnaires in a comprehensive survey, the research team initiated a meticulous process of data refinement. Through a rigorous preliminary analysis and strict on - site quality control measures, 6,847 substandard questionnaire responses were systematically excluded. These subpar entries mainly consisted of those with content losses, whether complete or partial. As a result, a refined dataset of 42,346 high - quality responses was obtained. Building upon this refined dataset, the research team further carried out a data return quality control procedure to ensure the accuracy and reliability of the data for subsequent analysis.

First, concentrate on seven sections within a specific block, including logical errors in the questionnaire and outliers. For example, a BMI over 50 or a scenario where there are grandchildren but no children reported. These situations necessitate a callback for verification. Using Python 3.3, fields identified as problematic were assigned a value of 1, while non-problematic ones received 0. Ultimately, 3,930 seniors were flagged for callback verification, The questionnaire achieved validity for a cohort of 3,281 elderly individuals. As a subsequent measure, through a meticulous review of audio recordings, 649 respondents who demonstrated a lack of due diligence in completing the questionnaire were pinpointed and subsequently excluded from the pool of invalid samples. Eventually, to ascertain the field assignment codes and utilize SPSS 27.0 for the purpose of database cleansing in Discussion Round 4. Determine 41,859 as the final sample. The sample distribution is shown in Figure 2.


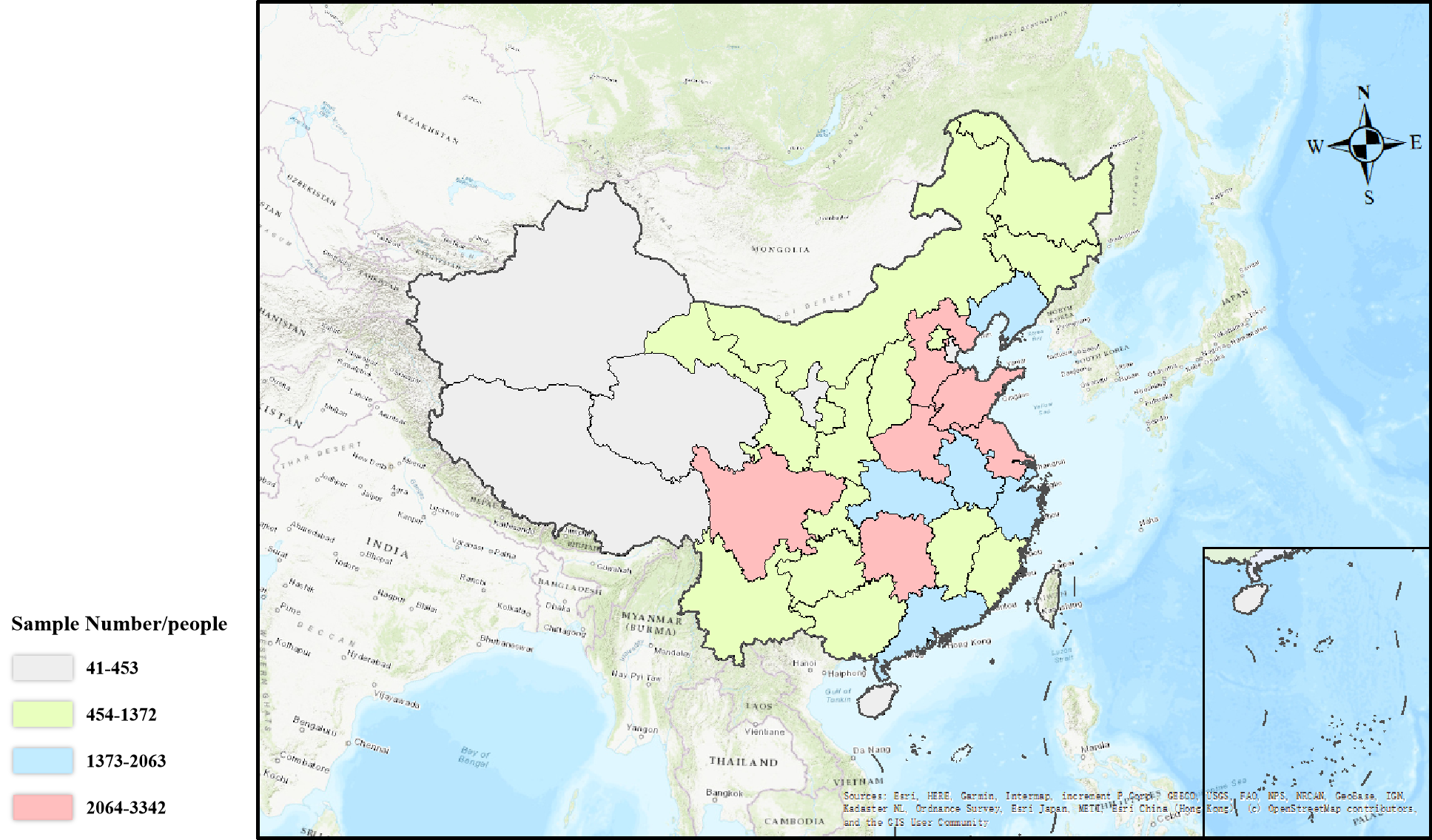


Figure 2. Sample Distribution of CAHS.
