# Supplementary material for: Association Between Intergenerational Relationship With Adult Children and Anxiety‐Depression Comorbidity Symptoms in Older Women in China: A National Study Using Latent Profile Analysis
Source: Depress Anxiety. 2026 Jun 15;2026:6040304. doi: 10.1155/da/6040304 (PMC13269846; doi:10.1155/da/6040304)
Supplement: Supplementary file 3 — Supporting Information 3 Additional File S3: Variance inflation factor (VIF) for covariates in the multinomial logistic regression model. [file DA-2026-6040304-s004.docx]

Additional File 4. Variance inflation factor (VIF) for covariates in the multinomial logistic regression model

| Variables | VIF | 1/VIF |
| --- | --- | --- |
| **Age (ref: 65-75 years old)** |  |  |
| 76-85 years old | 1.19 | 0.84 |
| > 85 years old | 1.18 | 0.85 |
| **Marital Status (ref: widowed)** |  |  |
| Non-widowed | 1.17 | 0.86 |
| **Education Level (ref: Illiterate)** |  |  |
| Literate | 1.14 | 0.88 |
| **Residence (ref: rural)** |  |  |
| Urban | 1.11 | 0.90 |
| **Chronic Disease (ref: no)** |  |  |
| Yes | 1.16 | 0.86 |
| **SRH** | 1.02 | 0.98 |
| **FCV** | 1.08 | 0.93 |
| **Mean VIF** | **1.13** |  |

Note. Note. VIF = variance inflation factor; 1/VIF = tolerance. FCV = frequency of children's visits; srh = self-rated health.
