# Supplementary material for: Association Between Intergenerational Relationship With Adult Children and Anxiety‐Depression Comorbidity Symptoms in Older Women in China: A National Study Using Latent Profile Analysis
Source: Depress Anxiety. 2026 Jun 15;2026:6040304. doi: 10.1155/da/6040304 (PMC13269846; doi:10.1155/da/6040304)
Supplement: Supplementary file 4 — Supporting Information 4 Additional File S4: Sensitivity analysis: Orthogonal polynomial multinomial logistic regression of comorbidity classes (moderate/high vs. low) on FCV—testing linearity assumption. [file DA-2026-6040304-s001.docx]

Additional File 3. Sensitivity analysis: Orthogonal polynomial multinomial logistic regression of comorbidity classes (moderate/high vs. low) on FCV – testing linearity assumption.

| Polynomial term | Moderate comorbidity vs. Low comorbidity | | | High comorbidity vs. Low comorbidity | | |
| --- | --- | --- | --- | --- | --- | --- |
|  | ****RRR (95% CI)**** | ****z**** | ****p-value**** | ****RRR (95% CI)**** | ****z**** | ****p-value**** |
| Linear (poly1) | 1.08 (1.04–1.11) | 4.55 | <0.001 | 1.18 (1.10–1.26) | 4.48 | <0.001 |
| Quadratic (poly2) | 0.96 (0.93–0.99) | –2.27 | 0.023 | 0.99 (0.92–1.07) | -0.21 | 0.833 |
| Cubic (poly3) | 1.00 (0.97–1.03) | –0.02 | 0.986 | 0.98 (0.92–1.05) | -0.65 | 0.513 |
| Quartic (poly4) | 1.01 (0.98–1.04) | 0.74 | 0.461 | 1.00 (0.94–1.07) | 0.12 | 0.903 |
| Quintic (poly5) | 1.00 (0.96–1.03) | –0.29 | 0.772 | 1.03 (0.97–1.10) | 0.96 | 0.337 |
| Sextic (poly6) | 1.06 (1.03–1.10) | 3.64 | <0.001 | 1.00 (0.94–1.07) | 0.13 | 0.898 |

Note. RRR = relative risk ratio; CI = confidence interval. Models were adjusted for age, education, residence, marital status, self-rated health, and chronic diseases. For moderate (vs. low) comorbidity, only the quadratic and sextic polynomial terms were significant alongside the linear term; the linear trend accounted for the predominant share of the association (moderate and high).
